# Multimodal MRI biomarkers and machine learning in neuromyelitis optica spectrum disorder: a review

**DOI:** 10.3389/fneur.2026.1751426

**Published:** 2026-08-18

**Authors:** Jiahui Zhang, Haoyou Xu, Min Zhao, Zequan Zheng, Yuanqi Zhao

**Affiliations:** Department of Neurology, Guangdong Provincial Hospital of Chinese Medicine, The Second Affiliated Hospital of Guangzhou University of Chinese Medicine, Guangzhou, China

**Keywords:** neuromyelitis optica spectrum disorder, multimodal MRI, imaging biomarkers, machine learning, differential diagnosis

## Abstract

Neuromyelitis optica spectrum disorder (NMOSD) is a relapsing inflammatory demyelinating disease of the central nervous system that can result in substantial cumulative neurological disability. Magnetic resonance imaging (MRI) plays a central role in lesion detection, differential diagnosis, and longitudinal assessment. However, conventional MRI may not fully capture subtle tissue damage and may show substantial overlap with related inflammatory demyelinating disorders in clinically ambiguous cases. Reliable imaging biomarkers reflecting disease burden, progression, and prognosis remain limited. This narrative review summarizes current evidence on conventional MRI features and advanced quantitative MRI biomarkers in NMOSD. We found that advanced techniques, including diffusion tensor imaging, morphometric analyses of T1 imaging, and resting-state functional MRI have revealed structural, microstructural, and functional abnormalities beyond lesions visible on conventional MRI. Furthermore, imaging-based machine learning applications have shown promising performance in differential diagnosis, particularly in distinguishing NMOSD from multiple sclerosis. However, findings across studies vary in the distribution, magnitude, and clinical relevance of reported abnormalities, likely reflecting differences in disease stage, acquisition protocols, and analytical methods. Additionally, most available studies remain limited by small cohorts, methodological heterogeneity, and insufficient external validation. Evidence for relapse prediction and treatment monitoring remains comparatively limited. Future research should prioritize multicenter prospective validation, harmonized imaging protocols, clearer separation of disease subgroups, and integration of imaging with clinical, serological, cerebrospinal fluid, and ophthalmic biomarkers before routine clinical implementation.

## 1 Introduction

Neuromyelitis optica spectrum disorder (NMOSD) is an inflammatory demyelinating disease of the central nervous system, most commonly associated with pathogenic aquaporin-4 immunoglobulin G antibodies (AQP4-IgG) (1). It is characterized by a relapsing disease course and severe attacks involving the optic nerves, spinal cord, area postrema, brainstem, and other central nervous system regions (2). In some cohorts, approximately 60% of patients have been reported to experience relapse within one year (3), which may lead to cumulative neurological disability, including visual impairment, motor deficits, and other irreversible sequelae. Therefore, early diagnosis, accurate risk stratification, and timely intervention remain critical challenges in NMOSD management.

In recent years, imaging and laboratory markers related to the diagnosis, disease monitoring, and prognostic assessment of NMOSD have continued to evolve. Magnetic resonance imaging (MRI), as a non-invasive and widely available tool, plays an indispensable role in lesion detection and longitudinal assessment (4). Nevertheless, MRI findings in NMOSD may overlap with those of MS, MOGAD, acute disseminated encephalomyelitis, and several non-inflammatory mimics such as vascular myelopathies. For example, dural arteriovenous fistula at the craniocervical junction may present with longitudinally extensive spinal cord lesions and has been misdiagnosed as NMOSD (5). Identifying specific and reproducible MRI biomarkers remains important for improving differential diagnosis and individualized disease assessment (Table 1).

**Table 1:** Conventional and advanced MRI features of NMOSD.

Image sequences	Injures location and characteristic
Conventional MRI (T1/T2-WI) Characteristics	Optic nerve: Often shows long-segment involvement, particularly in the posterior optic nerve, and may extend to the optic chiasm. Acute lesions may show optic nerve swelling and enhancement. Spinal cord: Lesions are often centrally located and may extend over at least three vertebral segments; shorter lesions may also occur. Bright spotty lesions, spinal cord swelling, and patchy or ring-like enhancement may be observed. Brain: AQP4-antibody enriched regions including area postrema lesion, periependymal brainstem. Acute lesions may show patchy, cloud-like, or linear periependymal enhancement, whereas resolution may be observed during follow-up.
Diffusion tensor imaging	Abnormal FA, MD, AD, or RD may be present in both conventional MRI-visible lesions and normal-appearing white matter. Occult changes frequently involve the optic radiations and corticospinal tracts. While lesion-specific and tract-specific patterns vary according to the tissue compartment and analytical method.
Three dimensional T1-WI	Lack of focal cortical lesions. However, focal rather than diffuse cortical atrophy particularly affecting the right middle frontal gyrus, postcentral gyrus cortex, thalamus, prefrontal GM, occipital cortex and bilateral cerebellar cortices.
Resting-state functional MRI	Region-dependent and bidirectional ReHo and ALFF abnormalities have been reported, with reductions mainly in cortical regions and increases in several subcortical and temporal regions, including the caudate nucleus and thalamus. Findings vary across anatomical regions, patient populations, disease stages, acquisition protocols, and analytical methods.

NMOSD, Neuromyelitis optica spectrum disorder; FA, Fractional anisotropy; MD, Mean diffusivity; ALFF, Amplitude of low-frequency fluctuation; ReHo, Regional homogeneity.

Advanced MRI analysis techniques, including voxel-based morphometry (VBM), surface-based morphometry (SBM), diffusion tensor imaging (DTI), and resting-state functional MRI (rs-fMRI) have developed rapidly and can detect subtle structural and functional abnormalities that may be invisible on conventional MRI (6). By quantifying gray matter atrophy, white matter (WM) microstructural damage, myelin-related changes, and functional connectivity alterations, multimodal MRI may provide further insight into the imaging characteristics and pathophysiological mechanisms of NMOSD.

Machine learning provides a data-driven approach for identifying complex patterns from high-dimensional clinical and imaging datasets and for developing predictive models. In neuroimaging research, machine learning has been increasingly applied to diagnostic classification, prognosis prediction, and treatment-response assessment (7). Imaging-based machine learning models may therefore offer additional value for NMOSD by integrating conventional MRI features, quantitative imaging biomarkers, and clinical information. However, heterogeneity exists across different studies such as differences in patient populations, imaging protocols, preprocessing strategies, and machine learning methodologies. The clinical applicability is still unclear (Table 2).

**Table 2:** Summary of imaging-based machine-learning and related imaging-biomarker studies in NMOSD.

Study & design	Cohort & serostatus	MRI modality & features	Model	Validation	Performance	Key limitations
A. Differential diagnosis (NMOSD vs. MS)						
Clarke et al. 2021 (64) (Front Neurol)—observational, multicenter cross-sectional comparison (Australia/NZ)	66 AQP4-IgG-positive NMOSD vs. 100 MS; MOG-IgG tested in a subset (48/66 NMOSD, 52/100 MS), all negative	Conventional MRI (brain, optic nerve, spinal cord); radiologist-scored qualitative pattern features (LETM, “bright spotty” lesions, Gd-enhancing cord lesions, bilateral optic nerve involvement, brainstem lesion site)	Decision tree	Applied to derivation cohort only; no resampling or independent validation reported	Precision 87.1%; 143/166 patients correctly classified (86.1%); AUC 0.874	Qualitative, rater-dependent scoring; no external validation
Huang et al. 2021 (66) (J Transl Med)—retrospective, single-center case–control	38 NMO vs. 78 MS (*n* = 116); AQP4/MOG serostatus NR	T1-MPRAGE, T2-WI (brain white-matter lesions); 1,118 radiomic features (intensity, texture, LoG, wavelet), multi-level selection	Multi-parametric multivariate Random Forest	10-fold CV in training set (*n* = 86) + internal hold-out test set (*n* = 30); no external cohort	ACC 87.1%; AUC 0.902; sensitivity 87.3%; specificity 86.9%	Single-center; compared against only 3 raters’ visual read; cross-scanner reproducibility untested
Ma et al. 2019 (67) (J Magn Reson Imaging)—retrospective, single-center case–control	77 NMOSD vs. 73 MS; serostatus NR	T1-WI/T2-WI-based ROI radiomics; 273 radiomic features (intensity, texture, LoG, wavelet) in ROI	Logistic regression (LASSO-selected features)	Internal train-test split; no external validation	AUC 0.882	Requires manual ROI segmentation; single-center; no external validation
Wang et al. 2020 (68) (Front Physiol)—retrospective, single-center case–control	41 NMOSD vs. 47 MS; serostatus NR	T2-FLAIR; automated end-to-end features (segmentation-based)	3D compressed CNN	Repeated five-fold cross-validation ×15	ACC 75%	Small sample; limited interpretability; segmentation errors may propagate
Kim et al. 2020 (69) (Front Neurol)—retrospective case–control	125 AQP4-IgG-positive NMOSD vs. 213 MS	FLAIR; automated, end-to-end deep features	CNN (3D ResNeXt-based)	Pre-specified internal hold-out test set (*n* = 135); train 45%/validation 15%/test 40% split	ACC 71.1%; AUC 0.82; sensitivity 87.8%; specificity 61.6%	Class imbalance; limited interpretability (black-box); single-center, single-ethnicity (Korean) cohort
Huang et al. 2022 (70) (Front Immunol)—retrospective, single-center, three-class diagnostic study	67 MS, 162 AQP4-IgG-positive NMOSD, and 61 MOGAD (*n* = 290)	Conventional multi-sequence brain and spinal cord MRI (axial T2-WI, coronal T2-FLAIR, sagittal spinal T2-WI); lesion-centered patches and automated deep features	MIL-CoaT transformer with brain–spinal cord fusion	Internal 4:1 development–test split (231/59); no independent external validation	Fusion model: three-class ACC 81.4%; one-vs-rest AUC 0.933 for MS, 0.942 for AQP4-IgG-positive NMOSD, and 0.803 for MOGAD	Retrospective single-center study; internal split only; acute attack/relapse imaging; some patients lacked paired brain and spinal cord MRI; no external validation
Seok et al. 2023 (71) (Sci Rep)—prospective, single-center diagnostic cohort	70 NMOSD vs. 86 MS; 66/70 NMOSD patients AQP4-IgG-positive; all patients MOG-IgG-negative	3D FLAIR registered to T1-WI; five selected axial slices concatenated as a 5-channel input; automated deep features with Grad-CAM visualization	Modified ResNet18 CNN (5-channel 2D input)	Group K-fold training/validation; repeated internal test sampling (15 baseline scans per group ×100); no external cohort	ACC 76.1%; AUC 0.85; sensitivity 77.3%; specificity 74.8%; PPV 76.9%; NPV 78.6%	Relatively small single-center cohort; no external validation; binary classification only; selected-slice input and variable disease phase may limit generalizability
Eshaghi et al. 2016 (74) (Neurology)—retrospective, 2-center case–control (Tehran + Padua)	50 NMOSD vs. 49 MS; 35/50 NMOSD patients were AQP4-IgG-positive (seronegative cases included)	3D-T1 structural MRI (cortical/subcortical volume, thickness, surface across 50 GM regions + deep GM nuclei) plus conventional T2/FLAIR-derived white matter lesion volume	Random Forest	Repeated random 50:50 train-test split, 5,000 repetitions, both centers pooled; no independent external cohort	ACC 74% (sensitivity 77%, specificity 72%) using all GM measures; ACC 80% (sensitivity 85%, specificity 76%) adding WM lesion volume	Predominantly structural imaging; moderate sample size; pooled-center internal validation only; no independent external validation
Yan et al. 2021 (75) (Front Neurosci)—retrospective, single-center case–control	36 NMOSD vs. 47 MS; serostatus NR	QSM; 874 QSM-derived radiomic features across 7 deep-GM ROIs	Logistic regression	5-fold cross-validation	Dentate-nucleus-only model: ACC 86.7%, AUC 0.902, sensitivity 85.1%, specificity 88.9%; best overall (hybrid radiomics + demographic): AUC 0.927	Single sequence; small single-center sample; top performance requires demographic data, not imaging alone
Liang et al. 2025 (76) (Sci Rep)—retrospective, single-center case–control	36 NMOSD vs. 56 MS; serostatus NR	rs-fMRI; multilevel RSFC + ALFF + ReHo features (AAL atlas, repeated with HOA atlas)	Logistic regression (best of SVM/LR compared)	7:3 train-test split, supplemented by 10-fold CV	ACC 90.9%; AUC 0.929; sensitivity 100%; specificity 75%	Small single-center sample; no external validation
Eshaghi et al. 2015 (53) (NeuroImage Clin)—retrospective, multimodal case–control	30 NMOSD vs. 25 MS; serostatus NR	Multimodal: 3D-T1, DTI, rs-fMRI, T2-FLAIR, T1-WI; cortical thickness/volume, FA, RSFC, WM lesion volume, cervical cord area (fused)	Multi-kernel learning for multimodal data fusion	10-fold cross-validation	ACC 88%	Very small sample (*n* = 55) relative to feature count; high overfitting risk; heavy multimodal acquisition burden limits feasibility
B. Exploratory imaging associations with disability and relapse burden						
Chien et al. 2019 (88) (Brain Commun)—cross-sectional exploratory study with healthy controls	40 AQP4-IgG-positive NMOSD vs. 31 healthy controls	Multimodal MRI, OCT, and clinical measures; total/deep GM volume, cortical thickness, spinal cord atrophy, retinal measures, and graph-theory network features	N/A—graph-theory association analysis, not a trained classifier	N/A	GM volume associated with relapse frequency and disease duration (*p* = 0.021, *p* = 0.011)	Cross-sectional exploratory design; small sample; associative rather than longitudinally predictive; no independent validation
Kim et al. 2023 (89) (Brain Imaging Behav)—retrospective, single-center cohort, no comparator	20 NMOSD; serostatus NR	DTI (whole-brain structural connectome); structural disconnection metrics (tractography)	Connectome-based predictive modeling (CPM)	Internal leave-one-out cross-validation	*r* = 0.506 with EDSS (*p* = 0.028); *q*^2^ = 0.274	Very small sample (*n* = 20); leave-one-out CV prone to overfitting; cross-sectional EDSS correlation, not a longitudinal prediction

NMOSD, neuromyelitis optica spectrum disorder; NMO, neuromyelitis optica; MS, multiple sclerosis; AQP4-IgG, aquaporin-4 immunoglobulin G; MOG-IgG, myelin oligodendrocyte glycoprotein immunoglobulin G; NR, not reported; ACC, accuracy; AUC, area under the receiver operating characteristic curve; CV, cross-validation; CNN, convolutional neural network; CPM, connectome-based predictive modeling; DTI, diffusion tensor imaging; QSM, quantitative susceptibility mapping; RSFC, resting-state functional connectivity; ALFF, amplitude of low-frequency fluctuation; ReHo, regional homogeneity; GM, gray matter; ROI, region of interest; EDSS, Expanded Disability Status Scale; LoG, Laplacian of Gaussian; FA, fractional anisotropy; MWF, myelin water fraction; RCT, randomized controlled trial; LR, logistic regression; SVM, support vector machine; LASSO, least absolute shrinkage and selection operator; LETM, longitudinally extensive transverse myelitis; Gd, gadolinium; FLAIR, fluid-attenuated inversion recovery; AAL, Automated Anatomical Labeling; HOA, Harvard-Oxford atlas; OCT, optical coherence tomography; MOGAD, myelin oligodendrocyte glycoprotein antibody-associated disease; MIL, multiple-instance learning; CoaT, co-scale conv-attentional transformer; Grad-CAM, gradient-weighted class activation mapping.

**Figure 1:**
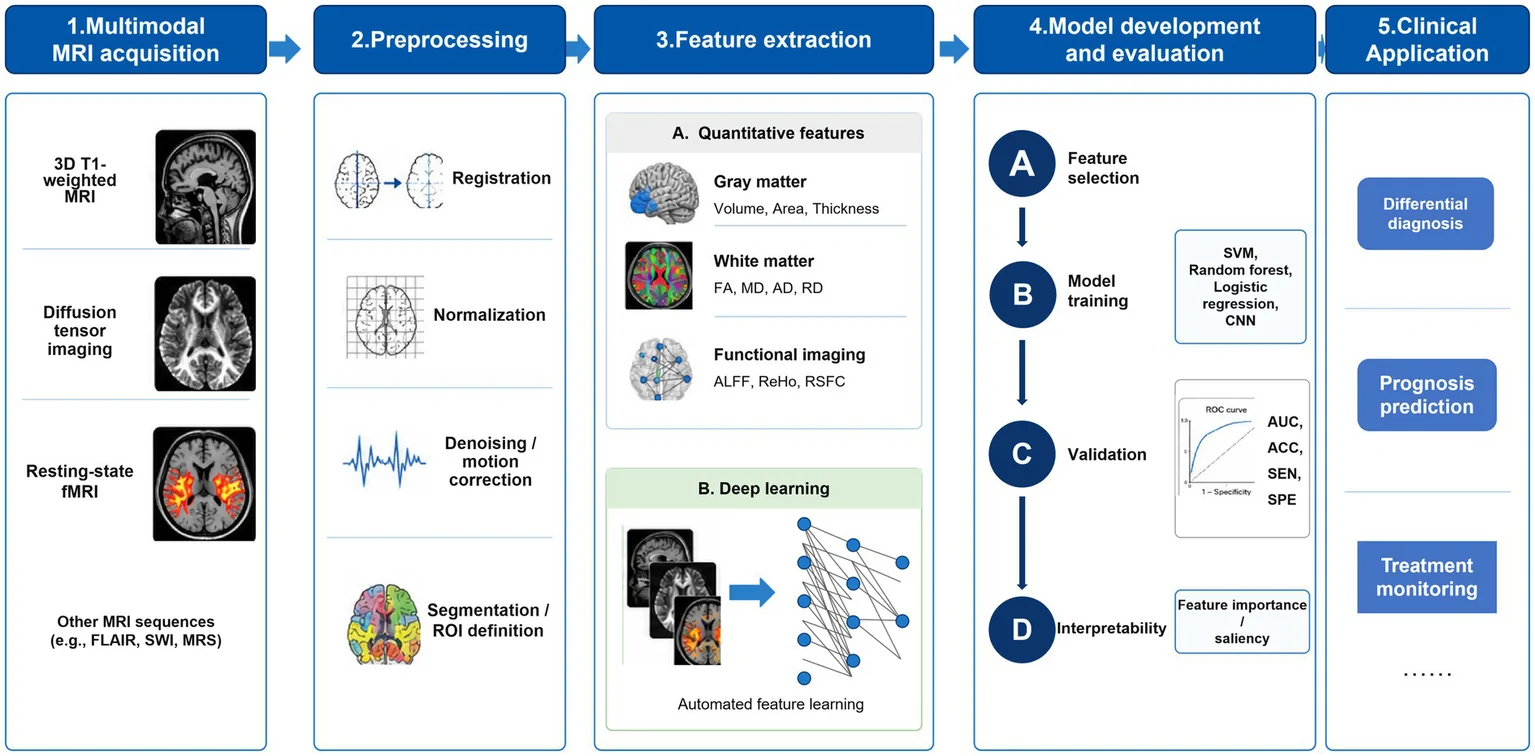
Schematic workflow of imaging-based machine learning in NMOSD.

This review aims to summarize current evidence on conventional MRI features, advanced quantitative MRI biomarkers, and imaging-based machine learning applications in NMOSD, with a focus on differential diagnosis, relapse risk assessment, treatment-response evaluation, and outcome prediction, so as to provide an integrated perspective for clinical application (Figure 1).

## 2 Conventional MRI features in NMOSD

Examination methods based on conventional MRI can provide preliminary qualitative analysis, such as the location, size, morphology and contrast-enhanced pattern of lesion but cannot yield a definitive diagnosis. Additional examinations, such as medical history, electrophysiological tests and cerebrospinal fluid analysis are still required. Even so, conventional MRI remains clinically valuable.

In AQP4-IgG-positive NMOSD, lesions frequently involve regions with high aquaporin-4 expression, including the periependymal regions, area postrema, brainstem, optic nerves, and spinal cord. Therefore, the aforementioned characteristic changes can also be observed on conventional MRI.

Brain MRI may demonstrate lesions in the dorsal medulla and area postrema, as well as periependymal regions surrounding the third and fourth ventricles. Acute lesions may show patchy, cloud-like, or linear periependymal enhancement, whereas partial or complete lesion resolution may be observed during follow-up. On spinal cord MRI, acute lesions are often centrally located on axial images and may involve the central gray matter. On sagittal images, the lesions frequently extend over three or more vertebral segments, although shorter lesions may also occur. Bright spotty lesions, spinal cord swelling, and patchy or ring-like enhancement may be observed. During the chronic stage, lesions may shorten or resolve, while severe attacks may result in cavitation and focal or longitudinal spinal cord atrophy. Optic nerve lesions commonly involve the posterior portion of the optic nerve and may extend to the optic chiasm. Acute lesions may show optic nerve swelling and enhancement, whereas persistent signal abnormalities or optic nerve atrophy may remain during the chronic stage (8, 9).

Currently, the widespread clinical application lies in the differential diagnosis between relapsing–remitting multiple sclerosis (RRMS) and NMOSD, which remains a critical clinical challenge due to substantial phenotypic overlap in disease manifestations. MS lesions predominantly affect periventricular regions (exhibiting Dawson’s finger configuration), juxtacortical zones, infratentorial structures, corpus callosum, and cortical gray matter. In contrast, NMOSD typically involves AQP4 antibody enriched regions, including periependymal regions surrounding the third ventricle and cerebral aqueduct, dorsal brainstem adjacent to the fourth ventricle, corticospinal tracts, and hemispheric WM (10). Longitudinally extensive spinal cord lesions involving at least three vertebral segments and optic nerve lesions involving more than 50% of the nerve length are important characteristic or supportive MRI findings of NMOSD, but they are not mandatory in all patients. Importantly, characteristic MS features—such as ovoid periventricular lesions, cerebellar peduncle involvement, and juxtacortical U-fiber lesions—are infrequent in NMOSD (11–13).

Although conventional MRI has improved diagnostic discrimination, the interpretation remains subjective and reliant on clinical expertise. Recent neuroradiology-focused reviews further emphasize that longitudinally extensive myelopathy and brainstem or middle cerebellar peduncle lesions are not disease-specific and should be interpreted within a broad differential diagnosis that includes MS, MOGAD, ADEM, inflammatory myelopathies, and non-inflammatory mimics (14, 15). Therefore, MRI findings should be interpreted together with clinical phenotype, AQP4-IgG and MOG-IgG status, cerebrospinal fluid findings, enhancement pattern, and lesion evolution, particularly in seronegative or clinically ambiguous NMOSD cases (16).

## 3 Novel MRI technologies and alterations in NMOSD

Conventional MRI has limited sensitivity for subtle abnormalities in normal-appearing tissue and provides little quantitative information on tissue integrity. Advanced quantitative techniques can complement conventional imaging by characterizing structural, microstructural, myelin-related, and functional alterations, offering further insight into tissue injury, though their clinical utility remains under investigation and methodological heterogeneity currently limits routine application.

### 3.1 White matter injuries in NMOSD

Diffusion tensor imaging (DTI), a non-invasive MRI technique based on diffusion-weighted imaging (DWI), evaluates WM microstructure by measuring water molecule diffusion patterns, revealing axonal damage and demyelination (17). The main parameters of DTI include fractional anisotropy (FA), mean diffusivity (MD), axial diffusivity (AD), and radial diffusivity (RD). FA reflects directional water diffusion coherence, with reduced values indicating impaired WM integrity and correlating with disease severity. MD measures average diffusion magnitude, increasing with edema or cellular necrosis. AD and RD capture diffusion parallel and perpendicular to axons, respectively, elevated AD suggests acute axonal injury and higher RD implies myelin disruption (18).

DTI studies have identified microstructural abnormalities in both visibly affected T2 lesions and normal-appearing white matter (NAWM) in NMOSD. Reduced FA and increased diffusivity have frequently been reported in pathways particularly vulnerable to NMOSD-related injury, including the optic radiations and corticospinal tracts. Abnormal diffusion measures have also been detected in periependymal regions and other areas anatomically associated with high AQP4 expression, including perichiasmal, periaqueductal, and periventricular regions, as well as thalamic pathways, even in the absence of corresponding abnormalities on conventional MRI (16). These findings suggest that conventional lesion assessment may underestimate the spatial extent of tissue injury in NMOSD.

Most studies confirm widespread FA reductions in NMOSD white matter and cervical spinal cord (19–25) in NMOSD. However, MD, AD, and RD changes show less consistency. Mechanistically, AQP4 antibody-mediated attacks increase membrane permeability, disrupting water molecule alignment and reducing anisotropy. Concurrent myelin damage and axonal loss further degrade fiber integrity, typically lowering FA while elevating MD, AD, and RD. However, early-stage patients without significant axonal injury may exhibit normal AD values.

Additionally, DTI has certain value in differentiating NMOSD from MS. Although substantial overlap of imaging features remains, patients with MS and NMOSD show different patterns of microstructural damage in T2 lesions and NAWM areas (26). The T2-visible lesions are substantially more abundant in MS and can dominate whole-brain WM comparisons (27). Chen et al. reported higher T2 lesion volumes in 14 of 20 evaluated WM tracts in MS than in NMOSD. Microstructural damage within lesions was also more severe in 13 tracts in MS (28).

Not only is total lesion volume larger in MS than in NMOSD, but lesion distribution also differs between the two disorders. In comparison with MS, tract-specific studies have reported DTI abnormalities in the fornix, corona radiata, inferior longitudinal fasciculus, optic radiations, and corpus callosum in MS, whereas the corticospinal tracts and optic radiations are frequently involved in NMOSD (24, 25, 29).

Finally, although the abnormal NAWM metrics could be found in both disorders across the evaluated tracts, the damage in MS is more extensive. Kim et al. previously demonstrated widespread occult NAWM abnormalities in NMOSD compared with healthy controls, but the abnormalities were more severe in MS, particularly for AD changes and involvement of association fibers such as the inferior cerebellar peduncle, external capsule, cingulum, superior fronto-occipital fasciculus, and uncinate fasciculus (26).

Taken together, DTI shows extensive microstructural damage in both visibly affected T2 lesions and NAWM in NMOSD. However, individual DTI metrics are unlikely to provide sufficient diagnostic specificity when used alone. Their greatest potential may lie in regional or multivariable imaging patterns combined with conventional MRI findings, clinical characteristics, and antibody status.

### 3.2 Gray matter structure abnormalities in NMOSD

NMOSD has long been considered an autoimmune disease that primarily affects the WM. However, because WM demyelination by itself cannot explain the full extent of clinical disability such as cognitive decline, renewed interest in gray matter (GM) pathology has been emerging (30). GM structural abnormalities in NMOSD have been investigated using double inversion recovery (DIR) imaging and high-resolution T1-weighted MRI combined with region-of-interest analyses, voxel-based morphometry (VBM), and surface-based morphometry (SBM). DIR imaging may improve the detection of cortical and juxtacortical lesions, whereas T1-weighted morphometric approaches permit quantitative assessment of regional gray matter volume, cortical thickness, and surface-based measures.

Previous histopathological analyses have revealed meningeal inflammatory infiltrates without concomitant cortical demyelination in NMOSD patients (31). A finding corroborated by 7 Tesla MRI studies also showed the absence of cortical lesions (32), suggesting that cortical lesions are usually not seen in patients with NMOSD. However, despite the lack of focal cortical lesions, patients with NMOSD exhibit structural alterations of GM in the cerebrum and cerebellum (33). Early studies identified focal GM abnormalities in NMOSD patients with unremarkable conventional MRI findings (34), while subsequent studies have demonstrated focal rather than diffuse cortical atrophy in NMOSD patients compared with healthy controls (HCs), particularly affecting the right middle frontal gyrus, postcentral gyrus cortex (35), thalamus, prefrontal GM (36), occipital cortex (37) and bilateral cerebellar cortices (38). Emerging evidence further suggests that GM volume loss is predominantly localized to sensorimotor, visual, and associated WM pathways (39–41). By contrast, some neuroimaging investigations failed to detect significant GM structural alterations (42). Differences in acquisition protocols, segmentation methods, patient characteristics, and statistical thresholds may contribute to considerable variability across studies.

Notably, recent research has revealed a subset of NMOSD patients manifest cognitive impairment (43), which is strongly associated with GM pathology. Current research has identified selective thalamic atrophy as a characteristic finding, particularly accentuated in cognitively impaired NMOSD patients relative to cognitively preserved counterparts (44). Intriguingly, cortical atrophy progression appears to be correlated with age and disease chronicity rather than acute inflammatory burden or lesion load (45), suggesting that neurodegenerative mechanisms may drive disability accumulation independently of inflammatory activity. These observations suggest that cortical neuroimaging abnormalities and cognitive dysfunction may arise from secondary neurodegenerative processes rather than primary cortical demyelination. The focal GM atrophy has been postulated to represent trans-synaptic degeneration secondary to optic nerve and spinal cord lesions (16).

Quantitative gray matter measures may also aid differentiation between NMOSD and MS. Compared with NMOSD, MS generally shows more extensive cortical and deep gray matter atrophy, particularly involving the thalamus, caudate nucleus, hippocampus and parahippocampal regions, and other limbic structures. These alterations have been associated with disability, cognitive impairment, and lesion burden in MS (46, 47). Conversely, NMOSD demonstrates limited deep gray matter involvement with preserved cortical architecture, potentially explaining its absence of progressive subtypes (48). However, most of these cohorts were predominantly or exclusively AQP4-IgG-positive; whether these findings extend to seronegative NMOSD or MOGAD remains largely unexplored.

### 3.3 Abnormal neuron activity in NMOSD

Neuronal and synaptic activity spontaneously generate low-frequency blood oxygen level-dependent (BOLD) oscillations (<0.1 Hz) during non-task conditions (49). Resting-state fMRI (rs-fMRI) detects BOLD oscillations and provides insights into functional neuropathology through three metrics: amplitude of low-frequency fluctuation (ALFF, neuronal activity intensity), regional homogeneity (ReHo, local synchronization), and functional connectivity (FC, interregional network correlations) (50). These biomarkers collectively characterize resting state cerebral functional architecture and interregional communication, providing critical insights into NMOSD pathophysiology (51).

NMOSD patients exhibit reduced ReHo and ALFF in cortical regions (precuneus, cingulate gyri, medial frontal gyrus) despite normal structural MRI (52). Independent component analysis (ICA) of network connectivity demonstrates diminished FC correlating with cognitive dysfunction (53), indicating widespread neuronal hypoactivity affecting perceptual, motor, and cognitive processing.

Paradoxically, concurrent ALFF elevations emerge in subcortical and cerebellar regions, including the caudate nucleus, parahippocampal gyrus, superior temporal gyrus, thalamus, and middle frontal gyrus (54, 55), while enhanced sensorimotor network FC inversely correlates with disability scores (56). This hyperactivation pattern may reflect compensatory neuroplasticity during motor or cognitive decline (51). This bidirectional pattern—regional hypoactivity alongside compensatory hyperactivation—suggests dynamic neural resource redistribution, where sustained hyperactivity may ultimately exacerbate neurodegeneration.

Functional MRI may also reveal group-level differences between NMOSD and MS. Although widespread network disruption has been observed in both disorders, more pronounced thalamocortical connectivity abnormalities have been reported in MS, potentially contributing to the greater cognitive impairment observed in some MS cohorts (57). Task-based fMRI during olfactory stimulation reveals differential activation: NMOSD patients exhibit reduced activation in the right superior temporal gyrus and left paracentral lobule, yet hyperactivation in bilateral insulae, hippocampi, parahippocampal gyri, left inferior orbital gyrus, superior temporal gyrus, putamen, and middle frontal gyrus compared with MS (58). These functional signatures provide potential diagnostic biomarkers. However, current evidence is based predominantly on small, single-center, and mainly AQP4-IgG-positive cohorts. Their reproducibility across centers and analytical pipelines, as well as their generalizability to seronegative NMOSD and MOGAD, remains to be established.

## 4 Application of machine learning in NMOSD

Machine learning is a rapidly growing field at present. Machine learning models incorporating diverse disease biomarkers demonstrate diagnostic and prognostic capabilities that can rival expert performance (59, 60). MR imaging biomarkers serve as critical tools for constructing models. In NMOSD, machine learning research mainly focuses on differential diagnosis with MS and prognostic prediction (53, 61–63).

### 4.1 Early differential diagnosis of NMOSD and MS

As mentioned above, the early identification of NMOSD from MS is of great significance. A model based on conventional radiological features achieved an accuracy of 87.1% and an AUC of 0.874 (64). Other region-of-interest-based approaches have reported accuracies or AUC values above 0.80 (65–67). Deep learning methods have also demonstrated potential, achieving accuracies above 70% (63, 68, 69), with convolutional neural network (CNN)-based image recognition attaining a remarkable accuracy of 98.8% in distinguishing MS from NMOSD (63). However, such exceptionally high performance should be interpreted cautiously: estimates from small, single-center, or internally partitioned datasets may be inflated by overfitting, class imbalance, scanner-specific characteristics, non-independent training and test samples, or information leakage.

Additionally, a transformer-based multiple-instance learning model integrating brain and spinal cord MRI differentiated MS, AQP4-IgG-positive NMOSD, and MOGAD with an overall accuracy of 81.4% (70). Similarly, a modified ResNet18 model using five selected axial FLAIR slices achieved an accuracy of 76.1% and an AUC of 0.85 for differentiating NMOSD from MS (71), with Grad-CAM indicating that WM lesions contributed substantially to classification. However, these models were developed retrospectively at a single center and evaluated using an internal train–test split, without independent external validation.

A subsequent systematic review and meta-analysis reported pooled accuracy, sensitivity, and specificity of 0.87, 0.86, and 0.84, respectively, for machine-learning and deep-learning models differentiating neuroimmunological diseases (72). However, substantial between-study heterogeneity, particularly for accuracy and specificity, limited the certainty and generalizability of these pooled estimates. A 2025 scoping review of deep-learning applications in neuroinflammatory diseases similarly found that computer-aided diagnosis and radiological segmentation currently dominate the field, whereas prognostic, functional, and treatment-related applications remain less developed (73).

Regarding novel MRI biomarkers, machine learning models using features of volume, thickness, and surface of 50 cortical GM regions and volumes of the deep GM nuclei achieved an accuracy of 80% for MS-NMOSD differentiation, identifying the thalamus as the most discriminative region (74). Quantitative susceptibility mapping (QSM)-derived deep gray matter susceptibility features yielded an accuracy of 86.7% and an AUC of 0.902 (75). Integrating rs-fMRI features with machine learning classifiers achieved 90.9% accuracy (AUC: 0.929) for NMOSD-MS discrimination (76). Multimodal MRI models combining deep gray matter volume, lesion volume, DTI, and functional connectivity metrics achieved a mean accuracy of 88% (53). Notably, visible WM lesions, fMRI, cognitive scores, and DTI received high model weights, while other modalities were assigned zero weights, underscoring the insufficiency of single-modal features and emphasizing the necessity of advanced multimodal imaging.

However, most models rely predominantly on cranial MRI, with limited incorporation of spinal cord imaging or optical coherence tomography despite their diagnostic relevance. Recent reviews have highlighted this gap, reporting that fusing brain and spinal cord MRI may improve discrimination and that pooled machine learning and deep learning models show favorable diagnostic performance for MS-versus-NMOSD classification, though these estimates should be interpreted cautiously given substantial differences in disease composition, imaging inputs, algorithms, and validation across included studies (72).

### 4.2 Exploratory imaging associations with relapse burden and disability

As noted above, a substantial proportion of NMOSD patients experience relapse within the first year (77). Early use of preventive immunotherapy to reduce the risk of additional relapses for high recurrence risk patients may be especially beneficial (78, 79). Although advanced neuroimaging has identified prognostic biomarkers in MS—such as paramagnetic rim lesions (PRLs), subacute enhancing lesions (SELs) (80, 81), and translocator protein (TSPO)-active lesions (82)—similar predictive biomarkers remain scarce in NMOSD. Clinical data-driven models have also shown limited potential in relapse prediction (83, 84).

Conventional imaging studies suggest associations between brainstem (particularly medullary) involvement and long-term disability (85–87), while reduced nucleus accumbens volume correlates with increased relapse frequency, and caudate nucleus atrophy correlates with disease duration (88). Regarding novel MRI biomarkers, only one study established connectome-based predictive modeling (CPM) using DTI to predict disability progression, associating frontoparietal WM disconnection with EDSS scores (89). However, the study estimated current disability rather than a prespecified future clinical outcome, even though a predictive modeling framework was used. At present, no validated imaging-based model has yet predicted future relapse or disability over a clearly defined prediction horizon.

The scarcity of prognostic models in NMOSD likely reflects the rarity of the disease, limited availability of large longitudinal cohorts, heterogeneity in relapse timing and preventive treatments, inconsistent imaging protocols and outcome definitions, and incomplete separation of AQP4-IgG-positive NMOSD, seronegative NMOSD, and MOGAD. A robust prognostic machine-learning pipeline should therefore use multicenter longitudinal cohorts, prespecified clinical outcomes and prediction horizons, harmonized MRI acquisition and preprocessing, explicit adjustment or stratification for antibody status and treatment exposure, and nested model development with temporal and independent external validation. Calibration, interpretability, and incremental clinical utility should also be assessed before clinical implementation. Given the value of global and regional brain atrophy measures in predicting disability progression and cognitive decline in MS, advanced MRI biomarkers may have potential for future prognostic modeling in NMOSD.

### 4.3 Treatment-response assessment and monitoring

Quantitative measures of tissue injury, including diffusion, myelin-sensitive, functional, and metabolic imaging markers may provide complementary information beyond conventional lesion assessment (90, 91). However, evidence supporting imaging-based machine-learning models for treatment-response assessment in NMOSD remains extremely limited. To date, no externally validated model has been established for monitoring therapeutic response or predicting treatment-related outcomes specifically in NMOSD. Nevertheless, a recent longitudinal study using machine-learning-assisted lesion segmentation found that inflammatory lesion volume increased significantly during remission in MS (3,493 → 4,430 mm^3^, *p* < 0.001), but remained relatively stable in AQP4-IgG-positive NMOSD (640 → 930 mm^3^, *p* = 0.129), suggesting distinct patterns of subclinical disease activity and potentially different MRI-monitoring strategies (92).

Additionally, a recent study in MS has suggested that deep-learning methods may identify contrast-enhancing lesions from non-contrast MRI and that myelin water fraction may serve as a marker of remyelination (93), particularly relevant given the association between NMOSD contrast-enhancing lesions and poor recovery (94). Thus, advanced MRI-based prognostic and therapeutic evaluation studies may emerge as future priorities in NMOSD. However, these findings represent indirect evidence and cannot be extrapolated directly to NMOSD because of differences in disease mechanisms, lesion characteristics, and treatment response. Prospective NMOSD-specific studies with standardized longitudinal imaging and predefined treatment-response outcomes are therefore required.

### 4.4 Methodological considerations

Advanced MRI biomarkers and machine learning approaches have shown potential for improving early differential diagnosis, disease assessment, and treatment monitoring in NMOSD. However, clinical implementation of advanced imaging biomarkers and machine learning models remains limited.

Firstly, the clinical applicability depends on robust validation, model interpretability, and generalizability across different scanners, protocols, and patient populations. However, most available studies are based on relatively small or single-center cohorts, with heterogeneous MRI scanners, acquisition protocols, feature extraction methods, and patient populations. Direct comparison across these studies remains difficult given differences in diagnostic criteria, antibody status, acquisition and preprocessing, feature selection, algorithm choice, and validation strategy.

Additionally, most available studies were retrospective cohorts, and relied on internal cross-validation rather than independent external validation, with limited assessment of calibration, interpretability, and generalizability to seronegative NMOSD, MOGAD, and atypical presentations, which may lead to overestimated performance and limited generalizability.

In addition, several models were developed in geographically and ethnically homogeneous cohorts, and their performance may not generalize to populations with different demographic, genetic, disease-subtype, and healthcare characteristics. External validation across ethnically and geographically diverse cohorts is therefore essential before clinical implementation.

Moreover, feature selection, preprocessing, and hyperparameter tuning should be performed exclusively within the training data or cross-validation folds to minimize selection bias and information leakage. In addition to discrimination metrics, future studies should assess model calibration and incorporate interpretability approaches, such as feature-importance analysis, SHAP values, or saliency maps, to determine whether predictions are based on clinically plausible imaging patterns.

Future studies should prioritize multicenter prospective validation, standardized MRI acquisition and post-processing pipelines, transparent handling of class imbalance, independent external validation with decision-curve analyses, and greater incorporation of spinal cord and optic nerve imaging, compared directly against established clinical, serological, and radiological assessment to determine whether machine learning provides reproducible, clinically meaningful incremental value.

## 5 Conclusion and limitation

Advanced MRI techniques have revealed structural, microstructural, and functional abnormalities beyond lesions visible on conventional MRI in NMOSD. Current imaging-based machine-learning evidence is predominantly focused on differential diagnosis, particularly the distinction between NMOSD and MS, whereas applications in relapse prediction, disability progression, treatment-response assessment, and therapeutic monitoring remain exploratory and lack adequate independent external validation. Accordingly, these tools should remain adjunctive and should not replace clinical assessment or antibody testing. Their clinical applicability depends on robust validation, model interpretability, and generalizability across different scanners, protocols, and patient populations.

The present review has two additional limitations. First, its technical coverage is restricted, as detailed discussion focuses mainly on three relatively established advanced MRI approaches, namely DTI, structural morphometric analysis, and resting-state functional MRI. Other potentially relevant techniques, including magnetization transfer imaging and magnetization transfer ratio, myelin water fraction imaging, diffusion kurtosis imaging, neurite orientation dispersion and density imaging, free-water imaging and other advanced diffusion models, quantitative susceptibility mapping and susceptibility-weighted imaging, arterial spin labeling and other perfusion MRI techniques, magnetic resonance spectroscopy, quantitative spinal cord MRI, and quantitative imaging of the optic nerves and optic chiasm, were not systematically reviewed. Future reviews could provide a more comprehensive synthesis of these modalities.

Second, the available literature does not sufficiently distinguish among AQP4-IgG-positive NMOSD, seronegative NMOSD, and MOGAD. These populations differ in pathophysiology, imaging patterns, clinical course, and diagnostic uncertainty, and many studies included predominantly AQP4-IgG-positive patients or did not clearly report serostatus. Therefore, the summarized findings may not be fully generalizable to seronegative NMOSD or MOGAD. Future reviews and imaging studies should pay greater attention to diagnostically challenging cases, including AQP4-IgG-negative or equivocal presentations, atypical optic neuritis or myelitis, and vascular, infectious, inflammatory, or autoimmune mimics.

## Glossary

NMOSD: Neuromyelitis optica spectrum disorder
MOGAD: Myelin oligodendrocyte glycoprotein antibody-associated disease
MRI: Magnetic resonance imaging
VBM: voxel-based morphometry
rs-fMRI: resting-state functional MRI
DTI: Diffusion tensor imaging
DWI: Diffusion-weighted imaging
SBM: Surface-based morphometry
FA: Fractional anisotropy
MD: mean diffusivity
AD: Axial diffusivity
RD: Radial diffusivity
GM: Gray matter
ROI: Region of interest
BOLD: Blood oxygen level-dependent
ALFF: Amplitude of low-frequency fluctuation
ReHo: Regional homogeneity
FC: Functional connectivity
MS: Multiple sclerosis
CNN: Convolutional neural network
QSM: Quantitative susceptibility mapping
CPM: Connectome-based predictive modeling
MWF: Myelin water fraction
MTR: Magnetization transfer ratio
MRS: Magnetic resonance spectroscopy
